# Department of Practice of Medicine

**Published:** 1894-10

**Authors:** Allen J. Smith

**Affiliations:** Professor of Pathology and Lecturer on Mental and Nervous Diseases in the Medical Department of the University of Texas, Galveston


### Current Medical Literature.

### DEPARTMENT OF GYNECOLOGY.

BY ALLEN J. SMITH, M. D.,
Professor of Pathology and Lecturer on Mental and Nervous Diseases in the Medical Department of the University of Texas, Galveston.

GASTRO-INTESTINAL HEMORRHAGE IN THE NEWBORN.—Dr. W. B. Konkle, of Montoursville, Pennsylvania (*Medical News*, April 21, 1894), after describing a case of gastro-intestinal hemorrhage which occurred the day following birth in a male infant of healthy antecedents, and without any perceptible taint, discusses the production of this somewhat common symptom. Leaving aside such cases as hæmophilia, syphilis, gastric ulcer, etc., he calls up the relation which tying the umbilical cord bears; this should have rather a depleting effect than a congestive one upon the portal circulation, but tends at once to increase the abdominal arterial circulation. If this increased arterial pressure were cause of the hemorrhage, the author argues that the effect should be immediate, not postponed, as is nearly always the case until hours or even days after the cord has been tied. Rather would he refer the phenomenon to the failure or proportionate closure of the ductus arteriosus and of the foramen ovale. When these fœtal openings close, proportionately, the opening up of the pulmonary circulation makes up for the engorgement of the abdominal arterial area, due to the tying of the umbilical cord; but if the ductus closes more slowly than the foramen, then the abdominal arteries already engorged from the closure of the artery of the cord will be further distended at the expense of the pulmonary circulation. If on the contrary the ductus closes much more rapidly than the foramen, the lungs will be subjected to an active hyperæmia. The latter, probably, does not often occur, or if it does, does not imply the same danger as abdominal hyperæmia; but the author, to prove its occasional occurrence, quotes a case of Dr. Connelly's, in which death from profuse pulmonary hemorrhage occurred within two hours after birth.

---

HYDROPHOBIA.—Dr. Hiram Corson, of Morristown, Pennsylvania, one of the oldest and most reputable physicians in the profession of that part of the country (*Medical and Surgical Reporter*, May 19 and 26, 1894), states his belief in elecampaine as a specific for the cure of hydrophobic poisoning. He quotes a number of instances of its successful employment in support of his position, among them two control experiments among cows, and one instance where men were the subject of such a control experiment. The two men in question, having been bitten by the same rabid dog, sought medical aid. The one was given elecampaine and failed to develop the disease; the other was treated with other remedies but died from hydrophobia. So much that is absolutely valueless has been recommended from time to time for the cure of hydrophobia, that one hesitates to accept anything that is not clearly proved. Yet the testimony which Dr. Corson has gathered is of sufficient importance to warrant a careful and thorough trial of the remedy. The method of administering is as follows: "To one and a half ounces of good sound elecampaine root, bruised in a mortar, add one pint of new milk, boil down to half a pint, strain off, and when cold take at a dose, in the morning, fasting. No food should be taken for from three to five hours afterwards. Repeat the dose on the third morning and again on the fifth. It seems quiet essential that the decoction should be made of milk. Half the above dose to child of twelve and proportionately to younger children."

---

ALCOHOLISM, TREATMENT AND CURE OF THE DISEASE.—Dr. C. F. Taylor, of Pueblo, Colorado (*Therapeutic Gazette*, April 16, 1894), regards alcoholism in its chronic manifestations as essentially a disease, to be treated rather as a disease physically and psychically than as a mere habit by psychical means alone. He advises that ordinary cases, permitting a moderate amount of alcohol as long as the disease continues (three or four days at most as a rule), nitrate of strychnia be given hypodermically three or four times daily, beginning with gr. 1-120 and increasing to gr. 1-30, until some physiological symptom appears, as chilliness or muscular twitchings, when the dose should be diminished for a few days. In doses of perhaps gr. 1-50 ordinary cases will stand three or four hypodermic doses daily for thirty or more days with no ill effects. In four or five days after beginning the patient commences to have an appetite for an eaily breakfast and starts to mend. At the same time along with the strychnine Dr. Taylor administers sulphate of atropine (gr. 1-200 to 1-50) until marked physiological symptoms are present; this, however, being common within a week, sometimes within three or four days or even earlier. It should be discontinued, or the dose diminished, when the dryness of throat, "squeamishness," dry, suffused eyes, and dilation of the pupil become evident. Along with this hypodermic dosage the writer gives, internally, digitalis, generally in a tonic mixture of fluid extract of cinchona, muriate of ammonia and digitalis or strophantus. The atropine is discontinued in about one week but the other two remedies are generally continued a week, or ten days more before further change is required. Then gradually dropping one dose of strychnine, a dose of chloride of gold and sodium (gr. 1-20) is gradually increased to gr. 1-10. Gold is also added to the tonic in suitable doses, taking care not to abuse the remedy lest irritation of the bladder and gastro-intestinal tract result. At the beginning of treatment a cathartic is given and the bowels generally kept from constipation. Where a headache is complained of or a resting potion desired, phenacitine and salol (gr. v–vx) each are given. Where the patient has delirium tremens, the treatment is postponed until the delirium is over. The author cautions that needles be not used indiscriminately, and that separate needles should be used in syphilitic cases. He claims 95 per cent. of cures in upwards of 300 cases.

---

CRAMP AND ANGINA PECTORIS (*Amer. Jour. Med. Sciences*, May, 1894).—For more than a century following Heberden (1768, *Trans. Coll. Physicians in London*, vol. ii, p. 64), angina pectoris has been considered by a large part, if not the majority of the profession, as caused by a spasm of the cardiac muscle, analogous to those often met in the limbs. There have been offered a number of reasons, however, why such a spasm is incomplete, to account for all the phenomena of an attack of angina. Dr. F. P. Weber, in a recent communication, speaks of the ordinary angina attack (following Huchard, "*Maladies des Coeur*," 2d Ed., Paris, 1893) as analogous to the condition described as "claudication intermittente" by Charcot and others. This latter condition was originally noted in horses, consisting of a sudden loss of power, accompanied with pain, anæsthesia of the limb affected, with sometimes rigidity. Presently this wears off, and the horse proceeds, only to have another and another attack if urged too rapidly. It has been found in these animals that there has been a closure of some form of the vessels supplying the affected part. A similar condition has been described in man. It is believed that this lack of power is due to insufficiency of the blood current, and that the pain is also the result of this ischæmia. Occasionally, probably from the insufficiency of the restricted blood supply to remove useless and irritative waste products, a cramp occurs; and this may be associated with the loss of power, pain, etc. Thus after or in great exertions the blood supply is insufficient to keep up the tone of the exercised muscles, and a temporary uselessness occurs. Often rigidity also occurs, but not necessarily. Sometimes the cramps occur without being preceded by any marked diminution of power, etc. So in disease of the vessels resulting in gangrene, pains are sometimes felt from the lack of nutrition to the sensory nerve endings; or even lack of power may occur, as premonitory of the oncoming gangrene. Thus it is believed that the ordinary angina, in which a loose, flaccid pulse is met, is really an instance of claudication due to the absence of sufficient blood, caused by coronary closure; the cramps which sometimes occur generally leading to a fatal termination, and the heart stopping in systole—the non-fatal attacks being unaccompanied by cramp, and only showing a lowered muscular power of the heart, together with pain.

---

ANTAGONISM OF ERYSIPELAS IN DISEASE.—Dr. J. F. Moran, (*Virginia Medical Monthly*, April, 1894), after recounting two cases of his own, one of pneumonia and the other of diphtheria, both of which seemed to have been rapidly and favorably terminated by intercurrent attacks of erysipelas, states that he has gone over the literature of such cases, and found numerous instances of the benign influence of erysipelas as an intercurrent disease. It was first observed in 1828, when the fact was clearly recognized that erysipelas accidentally acquired exerted a favorable influence upon the course of chronic lupus or eczema. In fourteen cases of diphtheria treated by inoculations with cultures of the microbes of erysipelas (no other treatment employed), they were, with two exceptions, attended by recovery. The two exceptions noted died before the erysipelas had time to develop. Besides the diseases noted, erysipelas has been known to exert a benign influence in pulmonary phthisis(?), malignant syphilis, gonorrhœa, and in carcinoma and sarcoma. Of the tumor cases recorded, one sarcoma and one carcinoma case died as result of the erysipelas. Three out of seventeen cases of carcinoma collected were permanently cured, and the rest were, with the one exception above noted, benefited. In nearly every instance, the tumor was not a primary growth amenable to treatment. The author's belief as to the nature of this antagonism is that the erysipelas stimulates the natural property of the cells of the body to the evolution of an antitoxine, in their endeavor to resist the invading toxine, and thus maintain the integrity of the whole body.

---

TYPHOID FEVER.—James D. Moran (*Virginia Med. Monthly*, April, 1894) remarks that calomel judiciously given during the prodromes and the first days of the pyrexia of typhoid fever has a beneficial effect upon the whole after course of the disease, even shortening its duration. He prefers sponging with alcohol and water to the use of the Brand method of cool baths, which, because of the difficulty and inconvenience of its execution, is often objected to by patients, and frequently fails to quiet the restlessness and delirium, where the earier and less disturbed method of sponging with dilute alcohol and cold applied to the head accomplishes more.

---

PALPATION OF THE APPENDIX.—Edebohls (*Amer.. Jour. of Med. Sci.*, May, 1894) writes of the facility with which the appendix vermiformis may be recognized by palpation through the abdominal walls. The examiner stands on the right side of the patient, and applies two, three or four fingers of the right hand, with the palmar surface held almost flat down, upon the abdomen at or near the umbilicus, drawing them toward the anterior superior spine of the right ilium. In doing so, the pressure must be deep enough to recognize distinctly, along the whole route traversed by the examining fingers, the resistent surfaces of the posterior abdominal wall and of the pelvic brim. Pressure short of this always fails to allow recognition of the normal or but slightly increased appendix, which, when normal, feels like a more or less flattened, ribbon-shaped structure; when its walls have been the seat of some inflammatory changes, it is more or less rounded and firm, and somewhat sensitive to pressure. The pulsation of the right common and external iliac arteries, easily and plainly felt, as a rule, is a good guide in searching for the appendix, which is generally found just outside these vessels. While marked displacements of the appendix may be a source of difficulty, the minor deviations, so often found, may readily be made out by the palpating fingers; and the fact that it lies back of the caput coli usually does not materially interfere, as this part of the gut is almost always collapsed. The writer asks for fair practice of this procedure, to verify his position as to its value in recognizing the condition of the appendix prior to operative measures for appendicitis.

---

PILOCARPINE IN ERYSIPELAS.—Dr. G. W. Barr (*Therapeutic Gazette*, May, 1894) publishes an article based upon his experience with forty cases, in which he corroborates the favorable report published in the same journal, two numbers previous, by Dr. Sallinger, in regard to the action of pilocarpine in erysipelas. The writer, following the instructions of DaCosta, gives an initial dose sufficiently large to produce a marked physiological action; and this is repeated in two hours, then not till six hours, and finally again in two hours. This method has been productive, in the hands of Dr. Barr, of happier results than the administration of the remedy at even and regular intervals. The success of the treatment, however, is very much greater in proportion to the precipitancy employed in commencing the use of the drug after the initial symptoms have appeared. A case of several days duration does not respond so readily. The only case out of the forty in which the treatment was not successful, was one of a week's standing before the pilocarpine treatment was instituted. The medication should be by hypodermatic methods, about one-sixth of a grain in the beginning. Convalescence, as a rule, sets in about twelve hours after the first dose.

