# Erratum

**Published:** 1894-10

**Authors:** 


**Erratum.**—Wrong head to Prof. Smith's Department. Discovered too late for correction.

